# Modeling the epidemiological history of plague in Central Asia: Palaeoclimatic forcing on a disease system over the past millennium

**DOI:** 10.1186/1741-7007-8-112

**Published:** 2010-08-27

**Authors:** Kyrre Linné Kausrud, Mike Begon, Tamara Ben Ari, Hildegunn Viljugrein, Jan Esper, Ulf Büntgen, Herwig Leirs, Claudia Junge, Bao Yang, Meixue Yang, Lei Xu, Nils Chr Stenseth

**Affiliations:** 1 Centre for Ecological and Evolutionary Synthesis (CEES), Dept. of Biology, University of Oslo, P.O. Box 1066, N-0316 Oslo, Norway; 2 School of Biological Sciences, University of Liverpool, Liverpool L69 7ZB, UK; 3 Department of Geography, Johannes Gutenberg University Mainz, 55099 Mainz, Germany; 4 Dendro Sciences Unit, Swiss Federal Research Institute WSL, Switzerland; 5 Dept. of Biology, University of Antwerp, Groenenborgerlaan 171 B-2020, Antwerp, Belgium; 6 Danish Pest Infestation Laboratory, University of Aarhus, Dept. of Integrated Pest Management, Skovbrynet 14, DK-2800 Kongens Lyngby, Denmark; 7 Key Laboratory of Desert and Desertification, Cold and Arid Regions, Environmental and Engineering Research Institute, Chinese Academy of Sciences, Lanzhou, 730000, Gansu Province, China; 8 State Key Laboratory of Cryspheric Sciences, Cold and Arid Regions Environmental and Engineering Research Institute, Chinese Academy of Sciences, Lanzhou 730000, China; 9 State Key Laboratory of Integrated Management on Pest Insects and Rodents, Institute of Zoology, Chinese Academy of Sciences, Beijing, 100101, China

## Abstract

**Background:**

Human cases of plague (*Yersinia pestis*) infection originate, ultimately, in the bacterium's wildlife host populations. The epidemiological dynamics of the wildlife reservoir therefore determine the abundance, distribution and evolution of the pathogen, which in turn shape the frequency, distribution and virulence of human cases. Earlier studies have shown clear evidence of climatic forcing on contemporary plague abundance in rodents and humans.

**Results:**

We find that high-resolution palaeoclimatic indices correlate with plague prevalence and population density in a major plague host species, the great gerbil (*Rhombomys opimus*), over 1949-1995. Climate-driven models trained on these data predict independent data on human plague cases in early 20th-century Kazakhstan from 1904-1948, suggesting a consistent impact of climate on large-scale wildlife reservoir dynamics influencing human epidemics. Extending the models further back in time, we also find correspondence between their predictions and qualitative records of plague epidemics over the past 1500 years.

**Conclusions:**

Central Asian climate fluctuations appear to have had significant influences on regional human plague frequency in the first part of the 20th century, and probably over the past 1500 years. This first attempt at ecoepidemiological reconstruction of historical disease activity may shed some light on how long-term plague epidemiology interacts with human activity. As plague activity in Central Asia seems to have followed climate fluctuations over the past centuries, we may expect global warming to have an impact upon future plague epidemiology, probably sustaining or increasing plague activity in the region, at least in the rodent reservoirs, in the coming decades.

See commentary: http://www.biomedcentral.com/1741-7007/8/108

## Background

Plague (*Yersinia pestis *infection) is among the most feared of diseases [1]. As a zoonotic disease, environmental factors shape plague epidemiology through effects on the main rodent hosts, the flea vectors, the bacterium itself and secondary hosts (other small mammals, domestic animals, and humans) and alternative vectors (lice and other flea species). On short time scales, plague activity in the established foci is mainly determined by the abundance of rodent hosts and vectors, but bacterial evolution can be rapid and respond to changes in host availability and susceptibility as well as transmission rate and even seasonality [2-4].

Despite being a far less prolific killer than ever-present diseases such as malaria, plague is believed to have caused at least two pandemics with major demographic impact. The first pandemic known to Western history, 'the Justinian plague', severely reduced the Mediterranean European population between 540 and 750 AD, decimating the capital Constantinople in 541-542 and dashing the last attempt to reunite the Roman Empire [5]. But from 800 at the latest, plague was seemingly absent in Europe for over half a millennium until the 'Black Death', which most likely spread out from Central Asia (traditionally suspected to have spread into Europe from the Black Sea port of Caffa, to which it had been brought by raiders or merchants from Central Asia [6]) and reduced the European population by about 40-60% after 1346 AD [7,8]. As in Justinian's time, it seemed to persist locally in Europe over the next few centuries before again fading away, first from rural areas, but persisting in some places into the early 19th century [1].

Even when absent from most records, *Y. pestis *was always present in its original habitats, and a so-called 'Third Pandemic' emerged from the Yunnan province of China in the early 1800 s. Although not causing devastation on the scales of 'Justinian's Plague' or the 'Black Death', some strains of *Y. pestis *have spread and persisted in rodent reservoirs from the western United States and India to Africa (in particular Madagascar) [9].

While the geographical origin of Justinian's Plague is unknown, the Black Death and Third Pandemic both probably emerged from the interior of Central Asia, where the plague bacterium seems likely to have evolved after diverging from its most recent extant relative, *Y. pseudotuberculosis*, and is also still endemic in large areas [5,8-11]. Between epidemics, human plague occurs almost exclusively as a result of contact with the wildlife reservoir, but human-to-human transmission (through lice vectors or droplets) may allow an epidemic to flare up over much larger areas outside those of the natural host ranges [7,12,13]. Primary human infections occur mostly through the bites of fleas that have fed on an infected animal (rodent or secondary host) or from eating or processing an infected animal.

**Figure 1:**
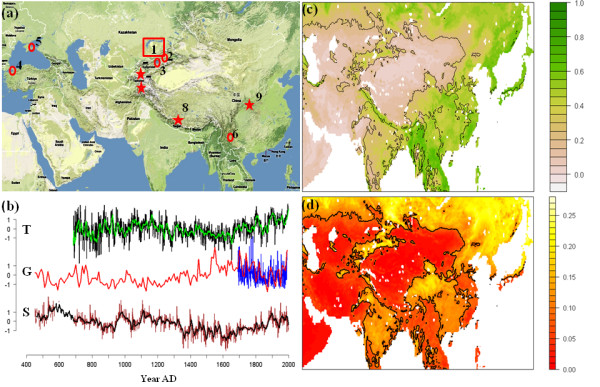
**Map and climate data**. **(a) **Map showing the PreBalkhash plague focus (1) and other locations mentioned in the text: the Almaty hospital (2); Lake Issyk-Kul (3); the city of Constantinople (4); the city of Caffa (5); the Yunnan province (6); the areas containing the dendrochronological sites (T) (7); the Guliya glacier (G) (8); and Wanxiang Cave (S) (9). **(b) **The climate proxy time series. For T and S the green and black lines respectively represent 10-year moving averages, for G the red line is the decadal reconstruction, while the blue line is the annual series **(c) **Average (Mar-Oct) NDVI for East Asia. Black contour lines correspond to interannual variability as shown in **(d)**, which represent the standard deviation of the annual mean for each pixel. We see that the most green (coastline broadleaf) and most dry (arid grass/shrubland) regions tend to have the least annual variability. Black contours indicate the mean.

Here we examine data from the Pre-Balkhash plague focus of southeastern Kazakhstan (Figure 1a), where the population densities of great gerbils (*Rhombomys opimus*), a major reservoir host species [10,14], their fleas and the presence of plague were continuously monitored from 1949-1995 [15,16]. The gerbil population densities vary considerably through multiannual fluctuations but are highly spatially correlated on large scales, at least up to the distances of more than 200 km covered by our field data. Their spatial distribution appears to be limited by soils suitable for burrowing, while their temporal density fluctuations are mainly influenced by an interplay between density dependence (which operates around the burrow systems as they disperse mostly at the local scale, i.e., <15 km per generation), and variability in vegetation cover (which is synchronous over larger distances due to spatially autocorrelated precipitation and temperature) [15,16]. The main sylvatic (i.e., wildlife) plague transmission vectors are fleas [17,18], and increasing vector abundance is probably one of the reasons why moist, relatively warm spring conditions tend to increase the prevalence of plague in great gerbils [12,16,19]. Great gerbils usually have few visible symptoms of plague infection, and only moderate increases in mortality [12,20], and there seems to be a critical threshold of host population density necessary for plague to persist in an area [10,12,21].

The region is fairly typical of Central Asia in having a highly continental, and mostly arid climate, with some moister and more productive areas near rivers and along the Himalayan foothills. Climatic variation may impact plague dynamics directly through host density fluctuations caused by variable food abundance and survival rates, through vector (flea) population growth and survival, through temperature effects on the bacterium itself and, most important, through the interaction of several factors. For instance, increased humidity would tend to increase host population growth (through food abundance) and flea population growth (through favoring survival of subadult stages as well as host densities) at the same time [7,16,19,22,23].

Thus, fluctuating weather conditions have been suggested to affect the dynamics of plague within its reservoirs and, through this, the number of human infections, while some little-known factors seem likely to limit the distribution of plague between large epidemics [15,16,19]. Here we pursue the link between climate and plague by using a set of recently published palaeoclimatic reconstructions that are the longest and most thoroughly verified high-resolution records available for Central Asia: two accumulation series from the Guliya ice core (G), signaling large-scale, low-frequency precipitation changes [24-26]; a stalagmite record from Wanxiang Cave, China (S), providing information on the strength of the east Asian monsoon and, conversely, the Siberian winter monsoon [27]; and a composite dendrochronological reconstruction from the Tien Shan and Karakorum Mountains (T) capturing summer temperature (and to some extent rainfall variability) [28,29]. For details, see Figure 1b, the Methods section and the supporting information (Additional file 1).

If the climate reconstructions capture variability relevant for the plague system, we should hypothesize that (1) the monitoring data will show evidence of climate forcing on sylvatic plague prevalence, and (2) this will be reflected in a further, independent data set on human cases of plague, collected by the Kazakh health department from 1904-1995. Most of these cases occurred prior to 1949, when plague monitoring and control began as part of Soviet-wide efforts, and antibiotics and insecticides became increasingly available [18,30]. Thus, a consistent and important climate forcing on sylvatic plague should be detectable in a statistical connection between climate and the independent human case data, allowing us to look for associations between climate fluctuations and human plague on scales much larger than previously considered, as well as connecting these associations with the long-term, large-scale behavior of the plague system.

## Results

**Figure 2:**
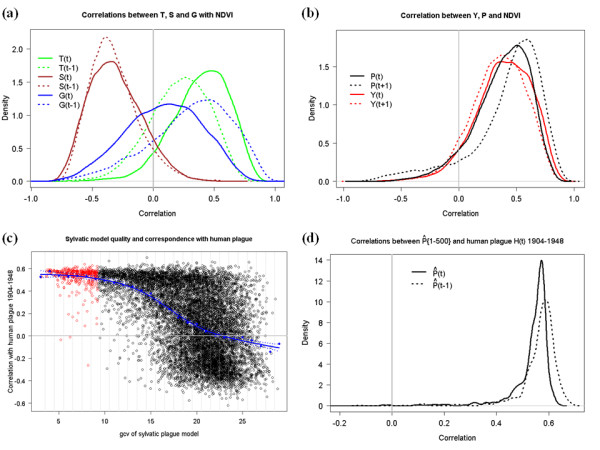
**Relations between climate and plague**. **(a) **Empirical distributions of the pixel-wise correlations between annual NDVI and the climate proxies. Only glacial accumulation is centered around zero for time lag 0, as accumulation rate the previous winter seems to be the relevant factor, while for T and S temporal autocorrelation gives both t = 0 and t = -1 significant relationships with NDVI. **(b) **Empirical distributions of the pixel-wise correlations between annual NDVI and sylvatic plague in the present (t = 0) and following (t = +1) year, both for observed (P) and estimated (Y) values. As for climate, plague seems non-randomly related to NDVI, both in the current and the preceding year. **(c) **GCV values for all 10^4 ^sylvatic plague models (eq. 8) vs. their correlation with human plague cases 1904-1948 (D). The 5% lowest-GCV models that form the estimate of climatically-forced sylvatic plague (Y) are highlighted in red. The blue dots show the mean D for each increment of GCV, connected with a (blue) trend line showing their close correlation (ρ = -0.99). **(d) **The empirical density distributions of D for the 5% (i.e., 500) best sylvatic plague models shown in red. They are very unlikely to be centered on zero, suggesting a nonrandom relationship between a climate-driven model's ability to predict sylvatic and human plague.

We find that for the period 1982-1998 (1991 for the ice cores), all climate indices have significantly nonzero average correlations with annual variations in primary production (measured as the Normalized Differentiated Vegetation Index, NDVI) over Central and East Asia (Figures 1c, 1d and 2a). Also, a significant relationship is found between annual variation in NDVI (using the most variable half of the pixels to rule out invariably green or dry broadleaf and desert pixels) and sylvatic plague abundance, P, in the same year (median pixel-wise *r *= 0.41, probability of mean *r *being zero <0.01) and also in the next year (median *r *= 0.50, *P *< 0.01) (Figure 2b). P is measured as the estimated annual product of gerbil density (animals per hectare) and the proportion of gerbils with active *Y. pestis *infection in the Pre-Balkhash 1949-1995 (see Methods). Associations with plague (P) are also found for the same-year tree-ring temperature index T (median *r *= 0.41, *P *< 0.01) and monsoon strength S (median *r *= -0.33, *P *< 0.01), and for the glaciological accumulation series (G) with a time lag of 1 yr (median *r *= 0.35, *P *< 0.01). Moreover, it is largely the same NDVI pixels that correlate with the climate indices as with plague (absolute pixel-by-pixel correlations between P_t _and NDVI give *r *= 0.74, *P *< 0.01 for both T_t _and S_t_, and *r *= 0.66, *P *< 0.01 for G_t-1_) (see Additional file 1 for maps). Thus, NDVI, plague prevalence in gerbils and the palaeoclimatic indices seem consistently interrelated (cross-correlated) for the available overlapping periods. As vegetation abundance is of direct importance as rodent food supply, and responds to moisture and temperature, which are also directly important for the fleas, bacteria and rodents, these associations suggests that the palaeoclimatic series reflect large-scale fluctuations in factors relevant for both the flea vectors and the rodent hosts of plague [12,15]. See Methods and Additional file 1 for further information.

**Figure 3:**
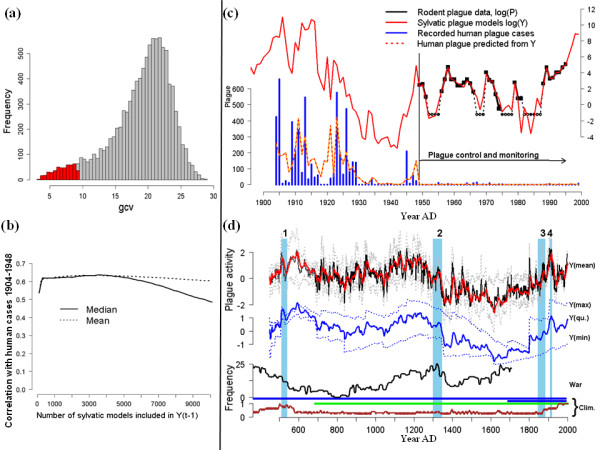
**Model results**. **(a) **The empirical distribution of GCV scores for the 10^4 ^climate-driven sylvatic plague models, the best 500 of which are marked in red. **(b) **The effect of increasing the number of models included in the sylvatic plague index Y on its correlation (D) with human plague 1904-1948. D is not sensitive to how many models are included, as long as the best 500 are. **(c) **The (log*_e_*) time series of sylvatic plague abundance (P, black; broken when no plague observed, i.e., *P *= 0, despite continued sampling of hosts), and the estimated plague abundance (Y, red line). The recorded human plague cases (blue bars) and the predicted human plague from Y (eq. 10, broken red-yellow line) are shown on a linear scale. **(d) **The black line shows estimated climate forcing on plague (Y) over the past 1500 years, with 95% quantiles in gray and multi-frequency (2-60 years) Gaussian moving average (red). The blue lines mark the long-term (2-400 years) multifrequency mean, maximum (upper broken line), minimum (lower broken line) and sum of minimum and maximum (solid line). The periods leading up to the Justinian plague (1), Black Death (2), Pandemic (3) and the Manchurian epidemics (4) are shaded in blue. The index (W) of conflict between Chinese and nomad societies is shown above the extent of the tree-ring index (T, green), the glacial series (G_ann _and G_dec_, blue), and the decadal coverage in the monsoon proxy (S, brown).

We therefore turn to links between climate, sylvatic plague activity and the independent data on human plague cases (Figures 2c, 2d and 3), initially for the period 1949-1995. The climate series show cross correlations with P (see Additional file 1), and of these, the tree-ring series (T) provides the highest correlation with plague prevalence (*r *= 0.45 at lag 0) as with NDVI, while the correlation with S is negative (*r *= -0.42 at lag 0). Low-frequency variations and delayed effects from climate are to be expected [15,19,21,31]. Hence, the number of plausible measures of climate exceeds what can reliably be corrected for, with regard to effects of multiple testing, without reducing the *P *value for acceptance below what is possible to achieve when temporal autocorrelation needs to be taken into account. Thus, instead of selecting one single "best" model, we fitted 10^4 ^variants of a statistical model with P as the response variable (see Methods, Figures 3c and 3d and Additional file 1), incorporating different measures derived from all three climatic indices (see Methods and Additional file 1). Each model's suitability for explaining observed sylvatic plague activity (P) from 1949-1995 was measured by its generalized cross-validation (GCV) score [32]. A substantial proportion of the large-scale sylvatic plague activity from 1949-1995 seems to be explicable through climatic factors, on average explaining 61% of the deviance, with the 5% models with lowest GCV explaining about 90%. This supports our first hypothesis. Also, the GCV scores are approximately normally distributed around a modal value of 22. However, a distinct "tail" of low GCV values strongly suggests that there exists a family of better-than-random models (Figure 3a; see also Methods and Additional file 1).

Moreover, as the climate proxies extend back in time, we compute the correlation, D, between a model's *predicted *sylvatic plague activity and the *observed *human plague cases 1904-1948, that is, before the sylvatic plague monitoring started. There is a clear correlation between D and a model's adequacy for explaining sylvatic plague (GCV) (ρ = -0.56, *n *= 10^4^, *P *< 0.001; Figure 3c). The *n *refers to the number of different ways of smoothing and lagging the climate data (see Supplementary information for further background). It suggests that models that are better at accounting for sylvatic plague activity 1949-1995 tend also to generate the better correlations between the sylvatic plague activity they predict and the human plague activity observed 1904-1948. Taking the mean correlation with human plague (D) per incremental increase in GCV underlines this, showing a very high correlation between GCV and mean D (ρ = -0.99, *n *= 27, *P *< 0.001) (Figure 3c).

**Figure 4:**
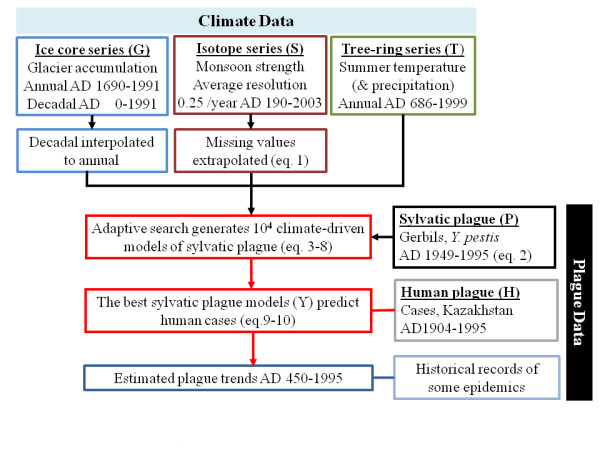
**Schematic overview**. The flow of information goes from the raw climate data, interpolated where necessary to keep a consistently annual scale over the whole period, to the models of sylvatic plague, and from there to their relationship with recorded human plague cases 1904 to 1949 and as a best estimate for plague activity over the last two millennia.

Focusing on the 500 models with the lowest GCV (best 5%), we find from the distribution of their D values that their predicted sylvatic plague activities consistently tend to correlate with human plague 1904-1948 (median *r *= 0.56, one-sample *t*-test *n *= 500, *P *< 0.001; Figure 3d). The temporal mean of these 500 predictions forms our 'best estimate' of climate forcing (Y) on sylvatic plague activity. It correlates closely with the number of human cases 1904-1948 in the same (ρ = 0.57) and in the following (ρ = 0.60) year, and this is not sensitive to the exact number of model variants included in Y (Figures 4a, b and 4c), as the poor (high GCV) sylvatic models cancel out, leaving the signal from the good (low GCV) ones dominant. Finally, taking temporal autocorrelations into account in a generalized additive mixed model (Methods and Additional file 1), we find that the sylvatic plague index, Y, is a significant predictor (*P *< 0.01) for human plague cases from 1904-1948, as well as for the whole period from 1904-1991 when taking the existence of the antiplague control program from 1949 into account as a bivariate factor (Figure 3c). As these human cases were recorded before the monitoring program started, they cannot have influenced the expectations or efforts of the people carrying out rodent monitoring. Thus, all these analyses are strongly suggestive of a consistent causal link between climate, sylvatic plague and human plague, and hence are consistent with our second hypothesis, that climate fluctuations affect the plague reservoir in a way that is decisive for the number of primary human infections.

Turning to much longer time scales, we first construct a binary vector, with 0 meaning no known large plague epidemic AD 450-2000, and 1 denoting suspected high plague activity leading up to a pandemic, namely the years 510-541 (prelude to the plague of Justinian), 1300-1347 (before the Black Death), 1845-1885 (the start of the Third Pandemic) and 1900-1920 (the Manchurian epidemics), the periods shaded in Figure 3d. The models whose predictions correlate the most with human plague in Kazakhstan 1904-1948 (D) also tend to have the highest correlations with this crude series of historical plague (ρ = 0.8, *n *= 10^4^, *P *< 0.01; see also Additional file 1).

Prior to the 16th century, the ice-core accumulation series (G) has only decadal resolution, and the monsoon index (S) has frequent missing values, which need to be estimated from its own decadal autocorrelation structure and the correspondence with the decadal ice-core accumulation (see Figure 3d and Methods). However, while such model and sampling error from several sources introduces large uncertainties, especially in the earlier parts of the reconstructions, there is no reason to believe that any of them introduces systematic bias. Hence, as a best estimate, the centennial scale picture emerging from the interplay between these climate factors is intriguing (Figure 3d). Despite the fact that the absence of tree-ring data deflates variability prior to AD 686, it suggests that the first (Justinian) pandemic started during a period of higher than median plague activity in wild rodents. Plague activity then declined slowly to below median, before rising in several smaller peaks from the beginning of the 12th century lasting until the onset of what became the Black Death around AD 1340. Afterwards, predicted plague activity declined to the lowest levels of the period and stayed well below the median until the time when plague again became evident in Chinese records and as the Third Pandemic emerged sometime during the late 18th and mid 19th centuries. During this period, about 2.5 million human plague cases were recorded over 18 provinces in China [33]. Furthermore, a short-lived peak coincides with the unusually virulent outbreaks of primarily pneumonic plague in Manchuria in 1910-1911 and 1920-1921 [34].

## Discussion

The Pre-Balkhash gerbil data cover about 2% of Kazakhstan. That the early 20th century human plague cases are nonetheless predicted so well on a larger (national) scale is probably partly related to the synoptic nature of the patterns involved. Indeed, climatic fluctuations synchronize gerbil abundance over large areas, which may itself be crucial to the spread of plague [15], as well as making the impact on humans greater simply by generating larger areas with high infection risk. Moreover, the relative explanatory power of a purely climate-driven model, such as that derived here, is likely to increase with the spatial scale over which it is applied, as the endogenous dynamics in otherwise uncoupled populations would then cancel out, increasing the relative strength of the larger-scale climate signal in the mean density. Human population density patterns (which reflect landscape productivity) also suggest that a large proportion of cases should occur in the southeast part of the country, in the region of the study site.

The impact of sylvatic plague on humans may operate through several scales and mechanisms [10,16,20]. First, it is obvious that more infected rodents and fleas are likely, everything else being equal, to translate into a greater number of humans coming into contact with the bacterium. Further, the more primary human infections there are, the more likely it is for an epidemic to start and for a particularly virulent mutant to establish in humans. However, everything may not be equal, as plague activity feeds back on itself through evolutionary processes. This may impact the resulting epidemiology when secondary hosts are infected and leads us to suspect that epidemics in naïve hosts could be more virulent when occurring after a prolonged period of high plague activity, even though (or indeed because) increased resistance in the primary hosts is also being selected for. This might suggest why the current pandemic is less virulent than those of centuries past.

No quantitative records of plague prior to 1904 are available for the region. But the 11th to 14th century period of high plague activity predicted by our study site coincides with plague outbreaks in Kaifeng and other parts of China from circa 1320 to the 1350 s [35,36] and notably with an outbreak near Lake Issyk-Kul (about 200 km from Pre-Balkhash; Figure 1) in 1338-1339 [37], the discovery of which suggested to archaeologists that the Black Death pandemic originated in this region [8].

The associations between the climatic indexes, predicted plague and NDVI suggest that periods of high plague activity should have a degree of correlation with increased precipitation and thus productivity in water-limited grasslands, as also suggested by earlier studies in Kazakhstan [16] and the USA [19]. Historical records reflecting the relative strength of the Han Chinese states versus neighboring pastoralist groups [38] are consistent with periods of high productivity preceding the two major plague epidemics (Figure 3d; see also Methods and Additional file 1). Resource scarcity is often assumed to be a primary driver of historical conflicts [39], and poor grazing conditions during cold periods probably forced Central Asian nomads south. However, territorial expansions and (successful) warfare depend not only on population size relative to its resources but also on the absolute size of the population from which armies can be recruited. We thus observe that the two main periods of border expansion, migration and warfare by Central Asian nomad pastoralists [38] found in Chinese records, and known in European history from the Hun invasions of the 5th century and the Mongol expansions of the 13th, are consistent with periods of high productivity in Central Asian grasslands having occurred prior to the great plague pandemics (Figure 3d). Also, Mongol expansions [35] might explain why plague appeared in Kaifeng (then recently occupied by Mongols) but not in Europe in the 1200 s, whereas during the 1300 s, after the Mongol conquest of Eastern Europe, plague appeared both in China and Europe [35].

Not much is known about the evolutionary rate of *Y. pestis *virulence in its natural reservoirs. But on a theoretical basis, we may suspect that periods of high plague activity (i.e., high host densities and transmission probabilities) select for more virulent types, and periods of low activity weed out strains killing the host too quickly [3]. Thus, it might be noteworthy that the centennial scale minima and maxima (i.e., averaging over moving windows of from 2 to 400 years in length) suggest essentially three periods of higher virulence having been selected for, and that these periods are consistent with the three large pandemic periods (Figure 3d). Moreover, depending on scale and the relative importance of virulence 'retreats' (i.e., plague minima) versus 'booms' (i.e., maxima), the Third Pandemic started with a markedly lower expected virulence than the other two and would therefore perhaps not have spread so far out of its original habitat had it not been for human activities. Interestingly, the largely pneumonic and virulent Manchurian epidemics of 1910-1911 and 1920-1921 [34] did appear during a short peak of high expected virulence.

There are some peaks that do not correspond to known plague events. These may point to weaknesses in the data or model or to unrecorded events (both likely, considering the paucity of data back in time), but it should also be noted that high plague activity in the reservoir is likely (as for other zoonoses) to be a necessary but not sufficient condition for large human outbreaks: plague in humans cannot be sustained without plague activity in the reservoir, but we do not expect every peak in rodents to generate a human epidemic. Moreover, there is no instantaneous link between Europe (or China) and the part of Central Asia with which our models are concerned. Thus, mechanisms for long-distance transmission by human or nonhuman hosts are also necessary, and our model of course cannot account for the local dynamics of plague foci persisting in Europe after the first waves of infection by some (presumably) virulent strain(s) of *Y. pestis*. However, as plague evidently has not been able to persist indefinitely in European hosts, it is notable that plague died off in rural Europe under periods of decreasing predicted plague peaks and virulence in Central Asia, though several European cities continued to act as independent plague foci for a considerable time, possibly due to the urban rodent reservoirs [8]. Thus, the apparent patterns are striking enough on several scales to suggest that the plague system has indeed been influenced by climate fluctuations over the past 1500 years and that this has had consequences for human history.

Even though the first, controversial isolates of *Y. pestis *DNA in European plague victims appear to have been satisfactorily replicated [1,40-45], there is ongoing debate about whether *Y. pestis *was a major cause of Justinian's plague and the Black Death [46]. Our findings suggest a consistent impact of climate on modern plague, are consistent with *Y. pestis *being present in medieval plague victims [40,44,45], and tentatively suggest that changes in host densities would select for fluctuations in pathogen virulence consistent with the historical trends. However, we must also caution that our climate-forcing models would arguably apply to other Central Asian zoonoses that could potentially have emerged and spread due to the same climate forcings.

Looking towards the future, plague is still a concern because of its potentially disrupting effects on local health systems and on international trade, and because of the risk of epidemics that are difficult to control, especially in dense human settlements with poor public health provisions [7,34]. While *Y. pestis *is currently not a major human pathogen, its status as a reemerging disease, combined with growing risks of antibiotic resistance [47] and its potential use as a bioweapon [48], makes plague a disease worth studying for its own sake. In addition, its reputation has motivated surveillance and research on scales that makes it a valuable model system for understanding zoonotic diseases in the past, present and future. The predicted regional climate changes are highly uncertain, but we note that increasing temperature (T) and monsoon (S) proxy evidences, and lower than average glacial accumulation, are related to higher plague estimates (Y). In other words, our results suggest that when the gerbils' dry habitats experience higher than average rainfall and warm springs, large plague outbreaks in gerbils, and high plague risk in humans, tend to follow. During the past decades, glacial accumulation (G) has been increasing and monsoon strength (S) has been about average, but the tree-ring temperature index, which is statistically the most closely and positively related to plague dynamics, has shown a rising trend which is expected to continue [29], and glacial accumulation may also fall as temperature rises [25]. Hence, predicted continuation of global warming associated with an increase of the hydrological cycle may enhance the probability of human plague outbreaks in Central Asia, especially if socioeconomic instability undermines the efforts of plague prevention in less developed though highly populated regions.

However, much work remains to be done. The epidemiological dynamics of the nonhuman hosts and the arthropod vectors are incompletely known, as are the differences between bacterial strains. The most common vectors have been studied in detail, but the sylvatic patterns are sometimes puzzling, suggesting some unknown mode of long-range dispersal or an unrecognized mode of persistence in the environment. Interactions with the habitat structure of the rodent host community may reflect some of the mechanisms through which climate, topography and vegetation (it is hard to avoid the impression that forest belts seem to act as boundaries) seem to form a barrier to plague occurrence, a barrier that seems to break down under some circumstances to allow the large-scale epidemics of history.

## Conclusions

The importance of climatic fluctuations in shaping human history is generally recognized [49], but such hypotheses are rarely evaluated statistically. Here, we assess the correspondence between multiple chains of evidence suggesting that large-scale climate fluctuations have important influences on the epidemiology of a reemerging disease of historical importance.

We do not imagine that our findings represent final, conclusive evidence that the historical pandemics known as the plague of Justinian and the Black Death were caused by the plague bacterium (*Yersinia pestis*), let alone that climate fluctuations were instrumental in the origin of these pandemics. But we do argue that climate fluctuations have important effects on modern plague epidemiology and that these effects are strong enough to be visible as consistent and nonrandom relationships between palaeoclimatic (proxy) data, remote-sensed variability in plant cover (NDVI), plague prevalence in an important wildlife host species (great gerbils), and human plague prevalence (number of reported cases) in the half-century prior to commencement of consistent public health efforts in the region.

This, we believe, shows that large-scale climate fluctuations have had, and likely will have, important consequences for wildlife plague prevalence and human health in vulnerable areas. Moreover, the correspondence between our climate-driven models of reservoir plague prevalence and historical trends in human plague occurrence are consistent with the view that large-scale plague epidemiology is linked to outbreak frequency and virulence in the core plague habitats of Eurasia, and that these habitats are affected by climate fluctuations. Such a view by no means suggests that human history is unimportant in explaining the great pandemics; on the contrary, for the climate forcings on Central Asian plague systems to have any effect on human populations as far away as China and Europe, long-range transmission must be possible and the receiving populations must be susceptible. Thus, our findings corroborate historical and archeological records suggesting that the Black Death at least originated somewhere in Central Asia and was transmitted (at least partially) through human activity to more densely populated areas.

Of more immediate concern may be that our findings also suggest that the partial discontinuation of plague surveillance following in the wake of the collapse of the USSR takes place at a time when current and near-future climate changes seem more likely to favor an increase than a decrease in plague activity.

## Methods

### Data

The ice-core data (G) are accumulation data from the Guliya ice cap of the western Kunlun Shan (35.31°N, 81.51°E), 6200 meters above sea level (Figure 1a). An annually resolved series (G_ann_) covers AD 1690-1991 with a ≤1 year dating error and correlates positively with annual precipitation and temperature to the west and south of the Himalayas [25,26]. Prior to AD 1690, only decadal scale resolution (G_dec_) exists. The annual ice-core series are moderately correlated (ρ = 0.34) with the decadal series, but taking the 10-year moving average of the annual series shows good decadal scale correspondence (*R*^2 ^= 0.83, *n *= 30, *P *< 0.01). We make a combined annual index (G) consisting of the mean of the annual series (when available) and the decadal series after being linearly interpolated to annual scale.

The stalagmite isotope data (S') are from Wanxiang Cave (33°19'N, 105°00'E) in the Gansu Province of China (Figure 1a), covering AD 190-2003. The record consists of 703 δ^18^O analyses with an average resolution of 2.5 years (Figures 1 and 4), less than 5-year errors in ^230^Th age, and less than 15-year errors from subsampling position (23). Analyses of historical records show that the δ^18^O records are negatively correlated with precipitation, and thus with the strength of the monsoon and related systems as far as glacier advances in the Swiss Alps [27]. Resolution is highest for the most recent periods, and during the model training period (1949-1995) only 1 year (1974) is unresolved. The missing data earlier in the monsoon index S are extrapolated from its own autocorrelation structure and the relation with the ice-core data (G_dec_), so that the estimates from the model in Equation (1) are substituted for missing values of S' to form the annual index S:

(1) $$S{'}_{t}=s_{0}+f_{1}(M_{mean}{\left(S',5,F\right)}_{t-1})+f_{2}(G_{dec,t})+\varepsilon_{t}$$

M_X_(y, z, g) denotes applying a function x on the time series y in moving windows of length z and type g (either Flat or Gaussian). The model is fitted using a generalized additive model (gam) with penalized regression splines. The parameters remain significant at the 5% level when taking autocorrelated normal residuals ϵ into account with a mixed-model (gamm) structure (see below), and give an adjusted *R*^2 ^= 0.48.

The dendrochronological time series (T) is a composite of 28 juniper tree-ring sites sampled over recent decades by several research teams in the Tien Shan and Karakorum mountains (Figure 1). These were found to have remarkably consistent growth patterns over different elevations and to reflect regional temperature and probably precipitation fluctuations [28]. The data are annually resolved over the AD 686-1999 period.

Thus, predictions before AD 1690 have a quasi-decadal resolution only, and prior to AD 690 they rely on only two of the three climate series.

The NDVI index is based on the differences between near-infrared and visible light reflected, giving an index of chlorophyll on the ground from almost zero (bare rock) to about 0.8 (rainforest). Our data are monthly composites from a 0.25 × 0.25 degree FASIR-corrected global data 1982-1998 [50]. Like regional temperatures [51], NDVI anomalies are connected over large areas, and we tested the palaeoclimatic indexes for correlation with annual mean value of each NDVI pixel 47-145°E, 13-54°N over the extended summer season (March-October) (Figure 2 and Additional file 1).

The sylvatic plague data are from monitoring of the Pre-Balkhash plague focus of southeastern Kazakhstan (748-788°E and 448-478°N) 1949-1995. The focus is divided into 10 × 10 km^2 ^sectors, four of which constitute a 20 × 20 km primary square (PSQ). Great gerbil density was estimated as the product of the relatively constant number of burrow systems per hectare (A) in each PSQ, *q *the proportion of burrows being inhabited (O) and mean number of gerbils per burrow (C) counted for 10 burrows per sector. Each season *v*, in spring (May-June) and autumn (September-October) [30], O and C were recorded for Q (1-78, median 54) PSQs. The data on gerbil plague prevalence were gathered independently from the gerbil population data, with a mean of 201 gerbils (min 1, max 4734) being trapped during spring and autumn. Of E trapped and examined gerbils, B tested positively for plague through isolation of *Y. pestis *from blood, spleen or liver smears. These data overlap spatially for about 79% of the population estimates at the PSQ scale. Gerbil mortality rates are not strongly affected by plague (recapture rates of seropositive animals ~84% that of seronegative over 3 years with bimonthly sampling), and gerbils only exhibit bacteraemia briefly and sometimes intermittently [10,12]. Hence, the data underestimate the sylvatic plague abundance (P) but are unbiasedly proportional to the following:

(2) $${\text{P}}_{\text{t}}=\;\sum \left({\text{B}}_{\text{v},\text{t}}{\text{E}}_{\text{v},\text{t}}{}^{-\text{1}}{\text{Q}}_{\text{v},\text{t}}{}^{-\text{1}}\sum \left({\text{O}}_{\text{q},\text{v},\text{t}}{\text{A}}_{\text{q}}{\text{ C}}_{\text{q,v},\text{t}}\right)\right)\;\;$$

The human plague data (H) are hospital records from Almaty, the former capital of Kazakhstan, where plague cases have been recorded annually since 1904. The number of cases drops sharply in the early 1950 s, when coordinated antiplague efforts were initiated. Owing to the enormous areas in question, the impact of these efforts on the sylvatic plague dynamics can be assumed to have been small; but the wide monitoring, public education and targeted application of insecticides in areas with domestic animals or human habitation seem to have successfully reduced the number of human cases (perhaps by an order of magnitude or more).

The data on conflicts between Han Chinese and Central Asian nomads are from Figure 2 in Fang and Liu [38], linearly extrapolated and smoothed over 30-year moving averages. The combined index W (Figure 3d) is the sum of the normalized series for war and migration frequencies (years with recorded battles or immigrations per 30 years), and Han/nomad administrative border at the 110°E longitude, measured as distance south of the 20°N latitude. The data are shown separately in Additional file 1.

### Plague activity model

There are several mechanistic links between weather fluctuations and plague prevalence, with covarying factors such as temperature and precipitation affecting host and vector population growth and density and through these higher-level effects such as contact and transmission rates. Hence, a traditional dynamic model would be hard-pressed to include all relevant factors and the effects of scale. We have therefore opted for estimating the net effect of climate through its many pathways through a more statistical approach rather than use a more explicit model whose many parameters cannot be established and thus easily could introduce an unknown bias. As *c *some of the climate effects are likely to operate at lower than annual frequencies (see Additional file 1) or with different effects, being multiplicative or limiting factors, the approach here is to let the effect of immediate value (or short-term average, to lessen the effect of dating uncertainty in the S and G series) be conditioned on the longer-term state for the two series with precise annual resolution for modern times (T and S). The long-term state can either be represented by the mean alone or in combination with minimum, maximum or variance for the time period in question. Extreme precipitation/temperature can be as important as the mean in their impact in a cool, arid environment. For the G series, preliminary analysis suggested that only the longer-term state was applicable, owing to lower temporal accuracy and resolution.

However, the possible number of ways to combine different time lags and smoothing filters for the climate variables becomes very large. Thus, conventional model selection procedures would suffer from massive problems of multiple testing and the existence of several statistically equally valid models. If the data transformations do nothing but randomly change signals unrelated to the response (plague), we would expect model explanatory capabilities to be normally distributed. But if there is a subset of climate variable transformations that reflect the time scales and parameters important for real biological processes, these models will be visible as outliers. To combine a good representation of the relatively rare better-than-random models with an unconstrained search of the parameter space, an adaptive element was combined with random permutations in model generation (see Additional file 1). We then see whether the performance of the climate-driven sylvatic plague models is nonrandomly related to the accuracy of their predictions for the independent human case data.

To combine a good representation of the relatively rare better-than-random models and an unconstrained search of the parameter space, an adaptive element was combined with random permutations in model generation: A moderately good model found by trial-and-error fitting was used as a first starting point. A random number from 1 to *n *of its *n *x-values (equations S3 to S7) were redrawn from their defined ranges (see Table 1), giving a new model. If this new model had a lower GCV score than its predecessor, its x-values were retained and used as the starting point for the next of the 10^4 ^iterations. There was also a 1 in 100 chance that the selection procedure would 'jump' to another of the best (i.e., lowest GCV) 1% of x-values, using these as a new starting point. Thus, the combination of full and partial replacement of variables and starting values allowed us to develop an adaptive search strategy that overrepresented good but rare model settings while retaining a comprehensive search routine that would not get 'stuck' in local optima.

**Table 1:** A summary and description of terms^a^

Label	Type	Description	Range/value	**Eq**.
x_1_	Variable	Type of moving window	Integer (1,..,3)	4
x_2_	Variable	Long-term Max/min/variance relative to mean	Sample (0,..,1), Prob (1,..,6)5^-1^	4
x_3_	Variable	Length of moving window	Integer (2,..,20)	4
x_4_	Variable	Gaussian or flat moving windows	Factor (True/False)	4,6,7
x_5_	Variable	Standard deviation of Gaussian moving functions	Uniform (0,4)	4,6,7
x_6_	Variable	Length of moving window	Integer (2,..,10)	4
x_7_	Variable	Gaussian or flat moving windows	Factor (True/False)	4,6
x_8_	Variable	Time lag for model variable	Integer (-2,..,0)	4
x_9_	Variable	Length of moving window	Integer (0,..,4)	5
x_10_	Variable	Type of moving window	Integer (1,..,3)	6
x_11_	Variable	Long-term Max/min/variance relative to mean	Uniform (0,..,1)	6
x_12_	Variable	Length of moving window	Integer (2,..,20)	6
x_13_	Variable	Length of moving window	Integer (2,..,10)	6
x_14_	Variable	Time lag	Integer (-2,..,0)	6
x_15_	Variable	Length of moving window	Integer (1,..,10)	7
x_16_	Variable	Max df used by interaction term smooth function	Integer (4,..,10)	8
x_17_	Variable	Max df used by single-variable smooth function	Integer (3,..,5)	8
T	Data	Composite tree-ring index; annual, normalized	AD 686 - 2000	3,4
S	Data	Isotope proxy monsoon index; normalized semi-annual	AD 450 - 2000	1,5,6
G_dec_	Data	Glacial accumulation; normalized, decadal, interpolated to annual	AD 450 - 2000	1,7
G_ann_	Data	Glacial accumulation; normalized, annual	AD 1690 - 2000	7
O	Data	Proportion of gerbil burrows occupied in the PreBalkhash focus	AD 1949 - 1995	2
A	Data	Number of gerbil burrows per hectare in the PreBalkhash focus	AD 1949 - 1995	2
C	Data	Number of gerbils per burrow in the PreBalkhash focus	AD 1949 - 1995	2
E	Data	Number of gerbils examined for plague in the PreBalkhash focus	AD 1949 - 1995	2
B	Data	Number of examined gerbils having plague infection	AD 1949 - 1995	2
P	Data	Abundance of sylvatic plague in PreBalkhash, Kazakhstan.	AD 1949 - 1995	2,8
${\hat{P}}_{i}$	Estimate	Estimated (fitted) values of P for each iteration i of eq. 8.	AD 450-2000	
d	Data	Presence/absence plague control	AD 450 - 2000	10
H	Data	Reported number of human plague cases from Kazakhstan	AD 1904 - 1995	10
Y	Estimate	Average predicted sylvatic plague level	AD 450-2000	9,10
NDVI	Data	Normalized Differentiated Vegetation Index	AD 1982 to 1998	
D	Estimate	Correlation coefficients for the relationships between ${\hat{P}}_{i}$ and H	AD 1904-1948	
W	Data	The index of conflict between Han Chinese and Central Asian pastoralists [38]	AD 450-1700	

^a^For x_1.17_, the ranges from which new values were drawn under model construction are given.

When fitting climate-driven models of sylvatic plague (equation 8), the transformed climate variables *z*_1_-*z*_5 _depend on the x-values for iteration *i*:

(3) $$\text{If}\left\{ \begin{array}{l} t\geq 686,\;z_{1,i,t}=T_{t}\\ t<686,\;z_{1,i,t}=t_{0}+f_{3}(z_{5,i,t}) \end{array}\right.$$

(4) $$\text{If}\left\{ \begin{array}{l} x_{1,i}=1,\;z_{2,i}{}_{,t}=({\text{x}}_{2,i}{\text{M}}_{\text{min}}{\text{(T,x}}_{\text{3},i}{\text{,x}}_{\text{4},i}{\text{,x}}_{5,i}\text{)}+\\ \;(1-{\text{x}}_{2,i}{\text{)M}}_{\text{mean}}{\text{(T,x}}_{\text{6},i}{\text{,x}}_{7,i}{\text{,x}}_{5,i}{\text{))}}_{t-x8,i}\\ x_{1,i}=2,\;z_{2,i,t}=({\text{x}}_{\text{2},i}{\text{M}}_{\text{max}}{\text{(T,x}}_{3,i}{\text{,x}}_{\text{4},i}{\text{,x}}_{5,i}\text{)}+\\ \;(1-{\text{x}}_{2,i}{\text{)M}}_{\text{mean}}{\text{(T,x}}_{\text{6},i}{\text{,x}}_{7,i}{\text{,x}}_{5,i}{\text{))}}_{t-x8,i}\\ x_{1,i}=3,\;z_{2,i,t}=({\text{x}}_{2,i}{\text{M}}_{\mathrm{var}}{\text{(T,x}}_{3,i}{\text{,x}}_{\text{4},i}{\text{,x}}_{5,i}\text{)}+\\ \;(1-{\text{x}}_{2,i}{\text{)M}}_{\text{mean}}{\text{(T,x}}_{\text{6},i}{\text{,x}}_{7,i}{\text{,x}}_{5,i}{\text{))}}_{t-x8,i} \end{array}\right.$$

(5) $$z_{3,t}=M_{mean}{\left(S,x_{9,i},F\right)}_{t}$$

(6) $$\text{If}\left\{ \begin{array}{l} x_{10,i}=1,\;z_{4,i}{}_{,t}=({\text{x}}_{\text{11},i}{\text{M}}_{\text{min}}{\text{(S,x}}_{\text{12},i}{\text{,x}}_{\text{4},i}{\text{,x}}_{\text{5},i}\text{)}+\\ \;(1-{\text{x}}_{\text{11},i}{\text{)M}}_{\text{mean}}{\text{(S,x}}_{13,i}{\text{,x}}_{7,i}{\text{,x}}_{5,i}{\text{))}}_{t-x14,i}\\ x_{10,i}=2,\;z_{4,i}{}_{,t}=({\text{x}}_{\text{11},i}{\text{M}}_{\max}{\text{(S,x}}_{\text{12},i}{\text{,x}}_{\text{4},i}{\text{,x}}_{5,i}\text{)}+\\ \;(1-{\text{x}}_{11,i}{\text{)M}}_{\text{mean}}{\text{(S,x}}_{13,i}{\text{,x}}_{7,i}{\text{,x}}_{5,i}{\text{))}}_{t-x14,i}\\ x_{10,i}=3,\;z_{4,i}{}_{,t}=({\text{x}}_{\text{11},i}{\text{M}}_{\mathrm{var}}{\text{(S,x}}_{\text{12},i}{\text{,x}}_{\text{4},i}{\text{,x}}_{5,i}\text{)}+\\ \;(1-{\text{x}}_{11,i}{\text{)M}}_{\text{mean}}{\text{(S,x}}_{13,i}{\text{,x}}_{7,i}{\text{,x}}_{5,i}{\text{))}}_{t-x14,i} \end{array}\right.$$

(7) $$\text{If}\left\{ \begin{array}{l} t\geq 1690,\;z_{5,i,t}=M_{mean}{\left(\frac{1}{2}(G_{ann}+G_{dec}),x_{15,i},x_{4,i},x_{5,i}\right)}_{t}\\ t<1690,\;z_{5,i,t}=M_{mean}{\left(G_{dec},x_{15,i},x_{4,i},x_{5,i}\right)}_{t} \end{array}\right.$$

which gives sylvatic plague model *i*:

(8) $$P_{t}=\exp\left[\begin{array}{l} z_{0,i}+f_{4}(z_{1,i}|z_{2,i},x_{16,i})+\\ f_{5}(z_{3,i}|z_{4,i},x_{16,i})+f_{6}(z_{5,i},x_{17,i})+\varepsilon_{i,t} \end{array}\right]$$

The maximum length of the moving time windows were set to 10 years for S and T, and 20 for G, as lower frequencies may not be reliable in a data series of 46 years. The regression splines (equation 8) were bounded upwards to 10 degrees of freedom for the surfaces (f_4_, f_5_) and to 5 for the spline (f_6_) to decrease the risk of overfitting with biologically implausible multimodal effects and penalized so that the automated selection on the generalized cross-validation (GCV) criterion could completely remove terms.

Thus, we obtain a time series of predicted values of the sylvatic plague models (${\hat{P}}_{i}$) for each iteration of the model construction (equations 3 to 8), and use the mean of the *n *best (lowest GCV) as a prediction of sylvatic plague abundance so that

(9) $${\text{Y}}_{\text{t}}=\;\frac{1}{n}\sum_{i=1}^{n}{\hat{P}}_{i,t}$$

and

(10) $${\text{H}}_{\text{t}}={\text{ d}}_{\text{t }}+\;f_{7}{\text{(Y}}_{\text{t}})+f_{8}{\text{(Y}}_{\text{t-1}})+f_{9}{\text{(Y}}_{\text{t-2}})+\varepsilon_{\text{t}}$$

The regression models (equations 2, 8 and 10) are fitted as generalized additive models (gams) with penalized regression splines, where the penalty removes terms from the model if unwarranted. f(X|Y, z) denote penalized thin plate regression splines for the effect of X and Y constrained to a maximum of *z *degrees of freedom. The error terms ϵ are assumed to have quasi-Poisson distributions (i.e., including a dispersion term) with serial autocorrelation, taking overdispersion and temporal autocorrelation into account when calculating (Bayesian) *P *values.

For further analysis, we use the mean predicted value of the *n *best (lowest GCV) models of sylvatic plague (Y_t_) as an estimate of sylvatic plague abundance and as a predictor of human plague cases.

All regression models are fitted as generalized additive models (gams) with penalized regression splines, where the penalty removes terms from the model if unwarranted. f(X|Y, z) denotes penalized thin plate regression splines for the effect of X and Y constrained to a maximum of *z *degrees of freedom. The error terms ϵ are assumed to have quasi-Poisson distributions (i.e., including a dispersion term) with serial autocorrelation, taking overdispersion and temporal autocorrelation into account when calculating (Bayesian) *P *values.

Throughout this paper, Spearman's rank correlation (ρ) is used when normality assumptions are not met for the Pearson's correlation (*r*). The software R was used for all modeling.

For further discussion and simulations and simulations regarding our statistical approach, please see Additional file 1.

## List of abbreviations

G_ann_: Glacial accumulation, annual; GCV: The generalized cross validation model selection criterion; G_dec_: Glacial accumulation, decadal; H: Reported number of human plague cases; NDVI: Normalized Differentiated Vegetation Index; P: Abundance of sylvatic plague; S: Proxy (isotope) monsoon index; T: Composite tree-ring index; W: Index of conflict between Han Chinese and Central Asian pastoralists; Y: Predicted sylvatic plague. (See Methods and Table 1 for further details.)

## Authors' contributions

All authors discussed the data, analyses, results or paper drafts. KLK did the modeling and analyses. MB, TBA, HV, HL, CJ, LX and NCS participated in the writing of the paper and provided data or background on the biology and epidemiology. JE, UB, BY and MY. provided climatological data and background.

## Supplementary Material

Additional file 1 **Supporting information containing additional figures**. Figures S1-S13. Click here for file
