# Supplementary material for: Modeling the epidemiological history of plague in Central Asia: Palaeoclimatic forcing on a disease system over the past millennium
Source: BMC Biol. 2010 Aug 27;8:112. doi: 10.1186/1741-7007-8-112 (PMC2944127; doi:10.1186/1741-7007-8-112)
Supplement: Additional file 1 — Supporting information containing additional figures. Figures S1-S13. [file 1741-7007-8-112-S1.DOC]

**Supporting Information** for "Modeling the epidemiological history of plague in Central Asia: paleoclimatic forcing on a disease system over the past millennium" by Kausrud, Begon, Ben Ari, Viljugrein, Esper, Büntgen, Leirs, Junge, Yang, Yang, Xu & Stenseth


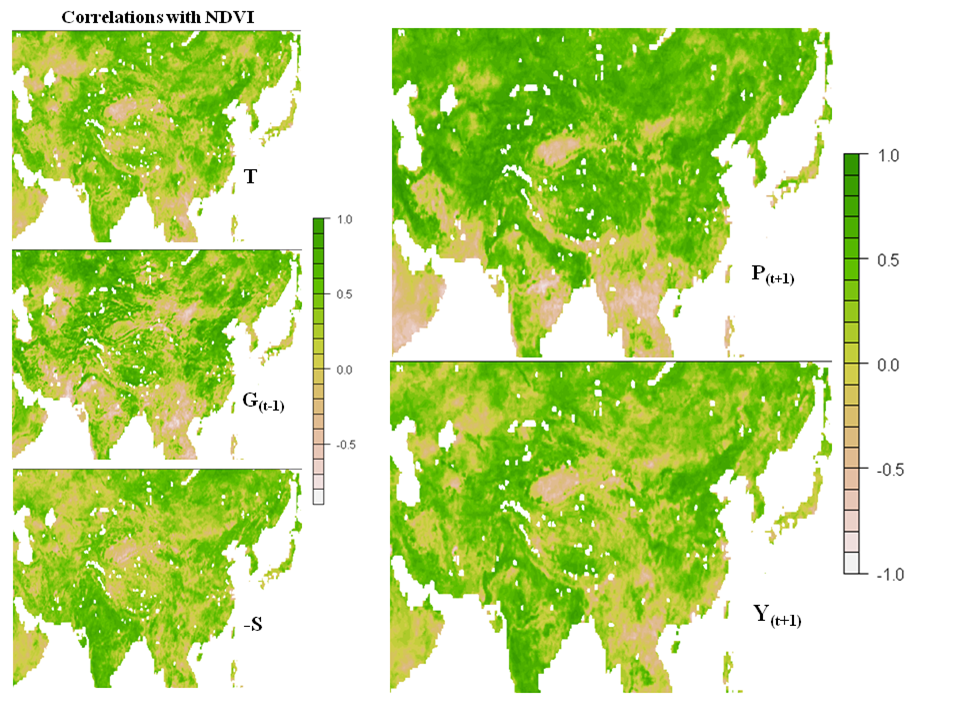


**Fig S1**: Pixel-by-pixel correlation coefficients between interannual variability in mean NDVI and the tree-ring index (T), the ice core series (G), isotope data (-S), plague abundance (P) and estimated plague forcing (Y).


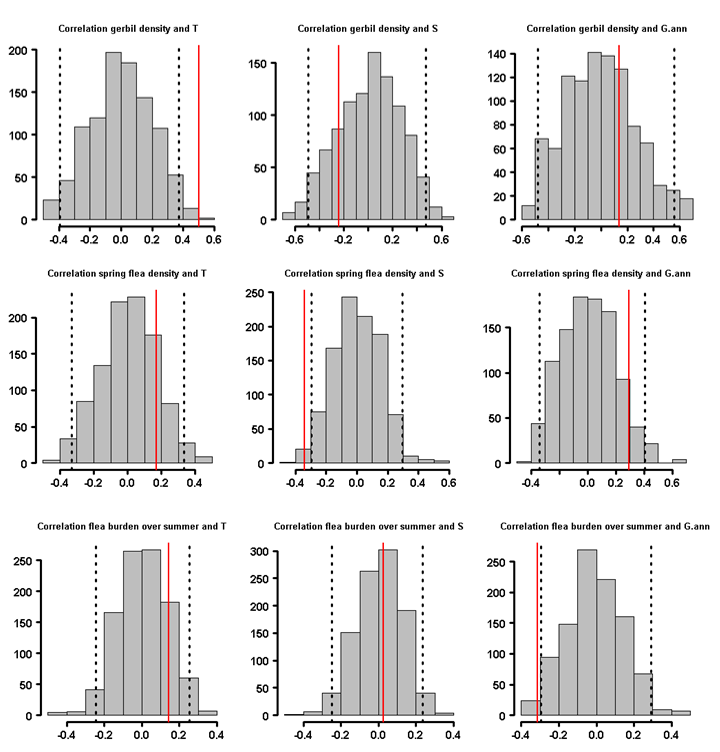


**Fig S2**: Randomisation tests of the correlation between the climate indices and the annual gerbil density (top) spring flea density (middle), and spring-to-fall flea population growth rate per gerbil (bottom). The red line shows the observed correlation, the dashed lines the 95 percentiles for the correlations expected from random permutations of the series. We see that the most robust correlation is between temperature (T) and gerbil population density (upper left corner).


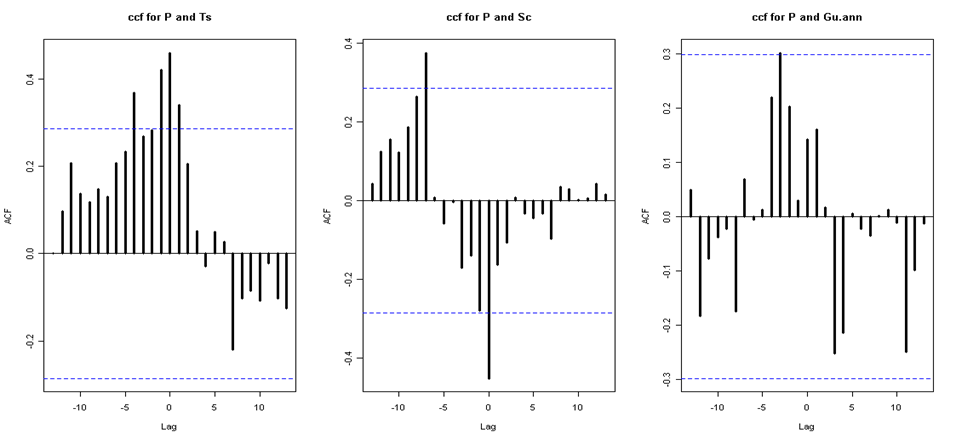


**Fig S3:** Cross-correlation functions for the paleoclimatic indexes (T, S, G_ann_) versus sylvatic plague abundance log(P+1).


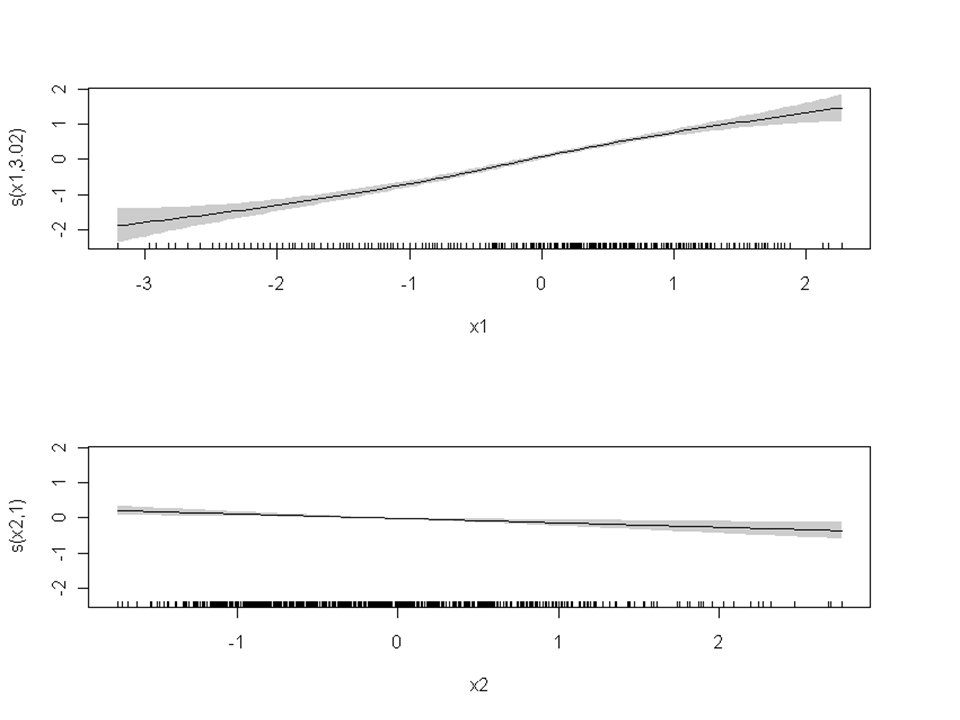


**Fig S4**: Effect plot for eq.1 (see main text), where x1 =$M_{mean}(S,5,F)_{t-1}$ and x2=G_dec_.


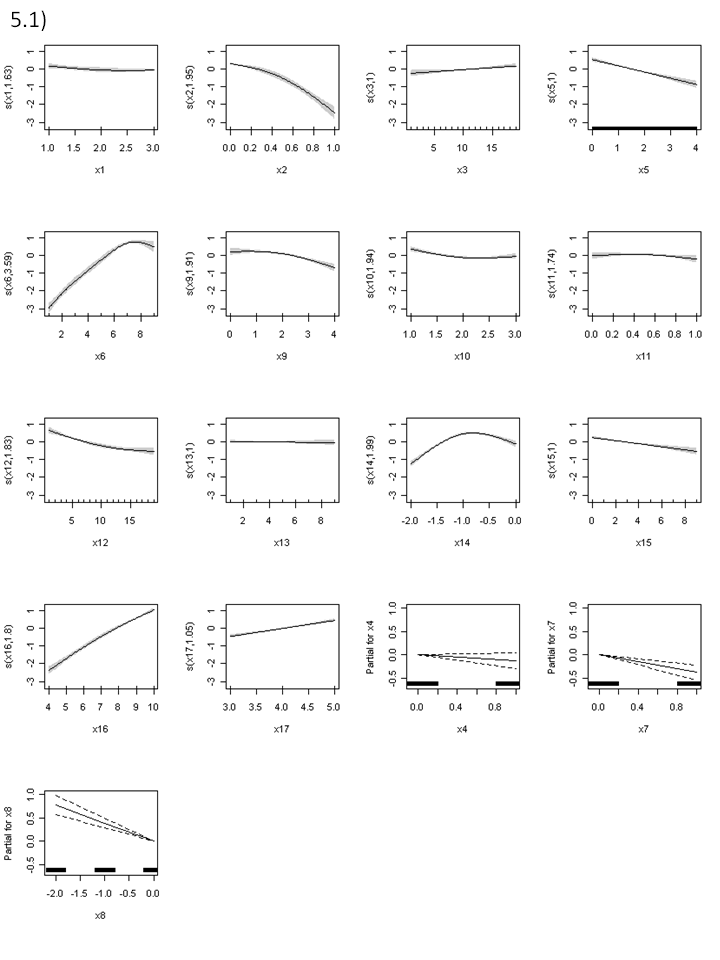


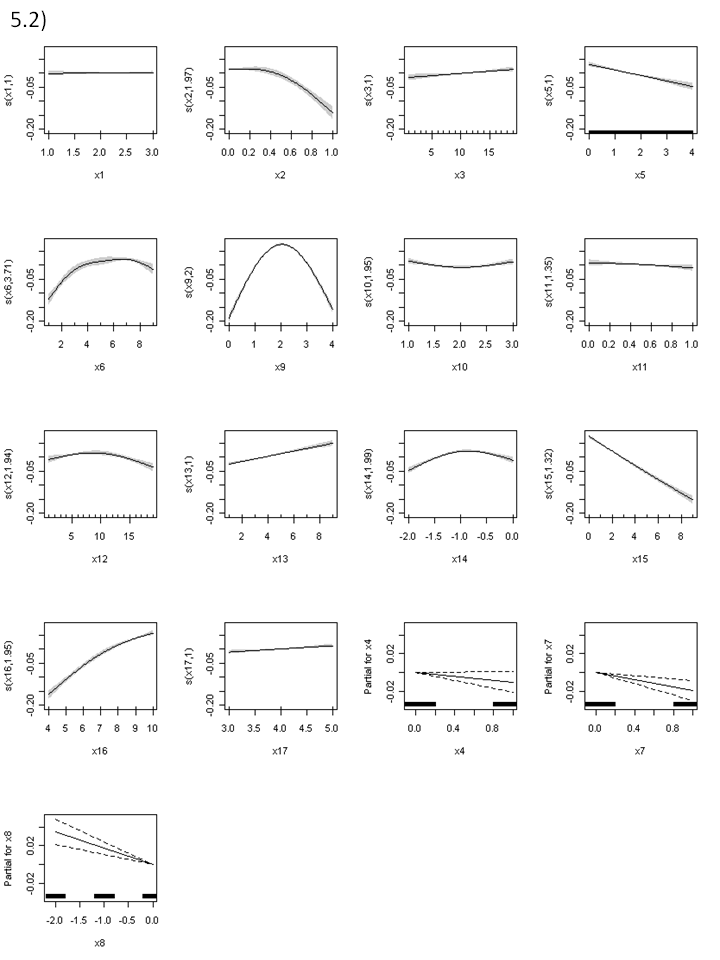


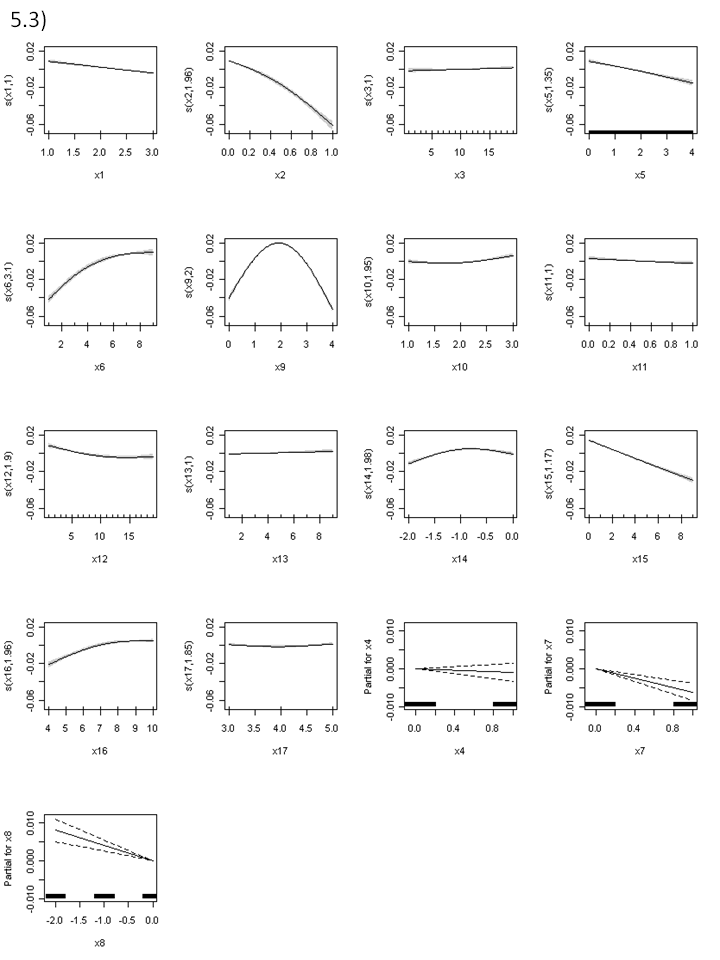


**Fig S5.1-5.3**: The effect of varying the 17 x-values (eqs. 3-7) over 10^4^ model iterations: 5.1 shows the effect on GCV, 5.2 shows the effect on correlation with human plague 1904-1948, and 5.3 shows the effect on correlation with the AD450-2000 binary historical epidemic-vector. The similarity between them is notable. Out of the 17 estimates, 14 (GCV, 5.1) and 15 (binary historical 5.3) share both sign and shape (positive/negative slope, convex/ concave/ linear effect) with 5.2 (observed human plague). A binomial tests for sharing sign this frequently (ignoring shape) suggests that even this would rarely happen by chance ( n=17, P=0.8, p<0.01) if the responses are unrelated and the effects of the explanatory variables would have been independent. As there may be hidden dependencies, the p-value should certainly not be accepted at face value, but the result is at least consistent with the different climate effects being similarly causally related to the responses.


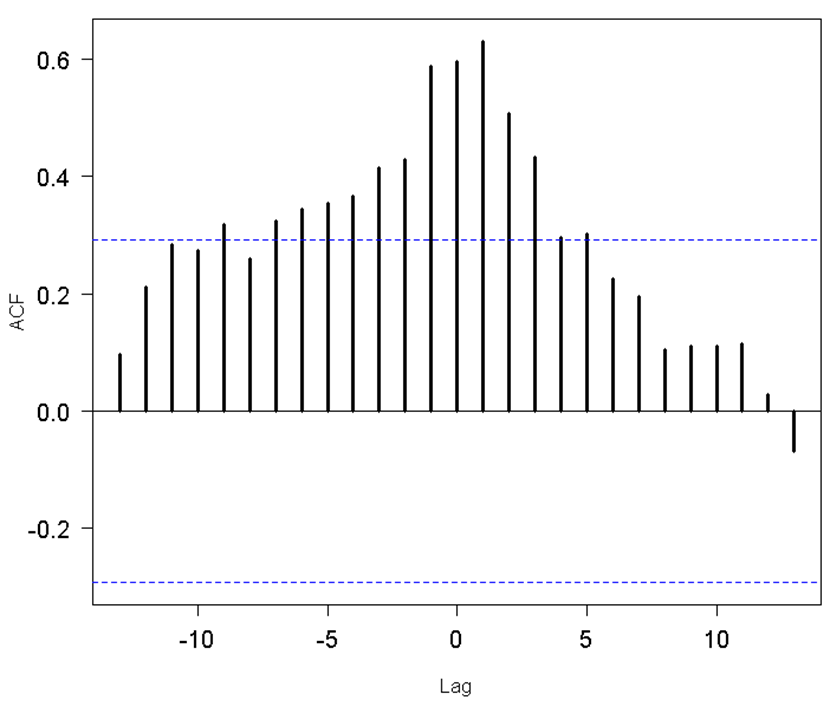


**Fig S6:** Cross-correlation functions for the predicted sylvatic plague index (Y) and human plague cases in Kazakhstan 1904-1948.


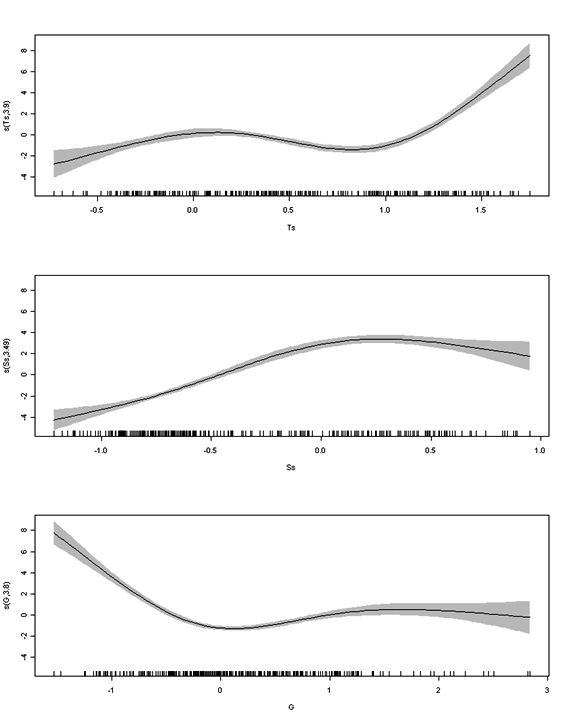


**Fig S7:** Effect plot showing how the climate indices (see main text) influence the final plague forcing estimate Y. T and S are decadal moving averages, and the model

Y_t_~f_10_(M_mean_(T,10,F)_t_)+f_11_(M_mean_(S,10,F))_t_+f_12_(G)_t_+ε_t_

explains about 85% of the deviance in Y.


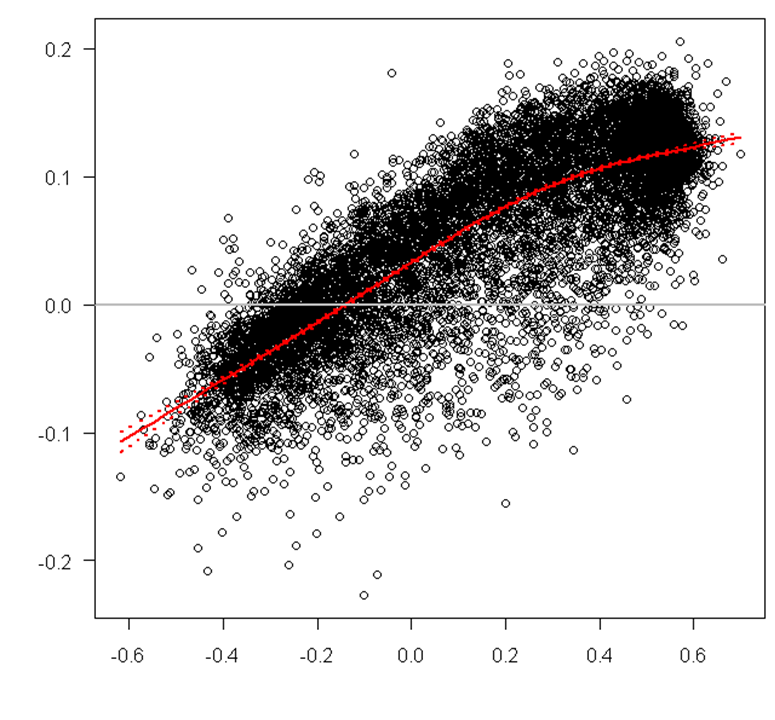


**Fig S8:**When we construct a binary vector with 0 meaning no known plague pandemic AD 450-2000, and 1 denoting periods of suspected high plague activity 510-541, 1300-1347, 1845-1885 and 1900-1920, we see that the models that make predictions correlating the most with human plague in Kazakhstan 1904-1948 (D) also tend to have the highest correlation coefficients with this crude series of historical plague (ρ=0.8, n=10^4^). Setting a p-value is tentative due to the multivariate autocorrelation structure, but taking this into account in a mixed-model setup still suggests that p<0.01.


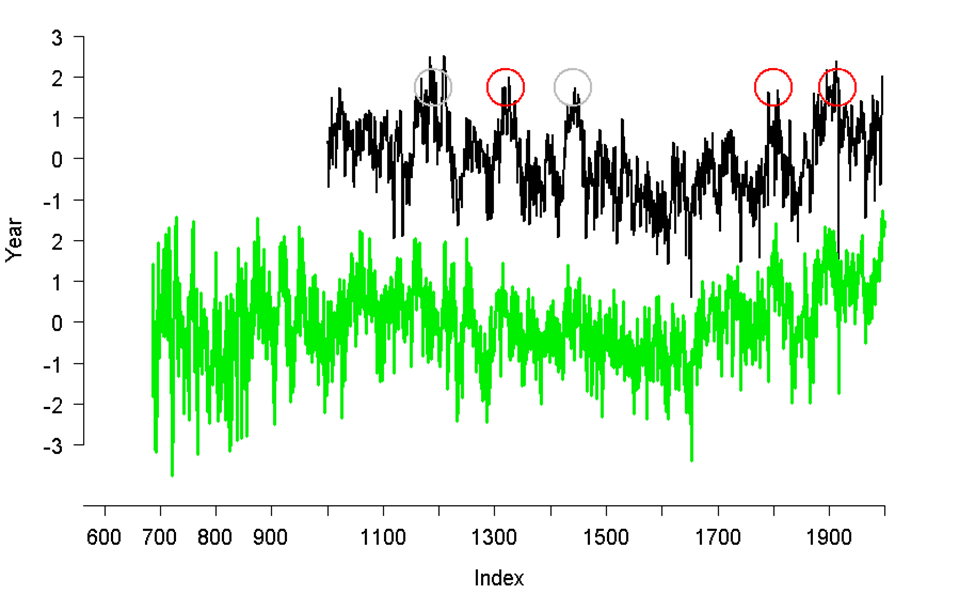


**Fig S9** The composite Karakorum –Tien Shan tree-ring series (Ts) used here is shown in green, a series using only Tien-Shan sites is shown in black. While this series has been shown to be less strongly correlated with temperature on a regional scale, it is interesting to note the peak growing conditions (probably reflecting temperature and precipitation to different degrees depending on what is the limiting factor at any given point in time) that occur in the early 1300's, 1800's and 1900's, (red circles) corresponding to known periods of high plague activity in the region. The two peaks in grey circles do not match particular plague periods known to history, unless the last one (1400's) is reflected in the plague waves following the Black Death. The first one was during a period of little contact between Europe and Central Asia, but the good growing conditions do correspond to the period when the Mongols evidently achieved population numbers great enough to initiate an empire and start a period of conquest under Genghis Khan in the 1200's.


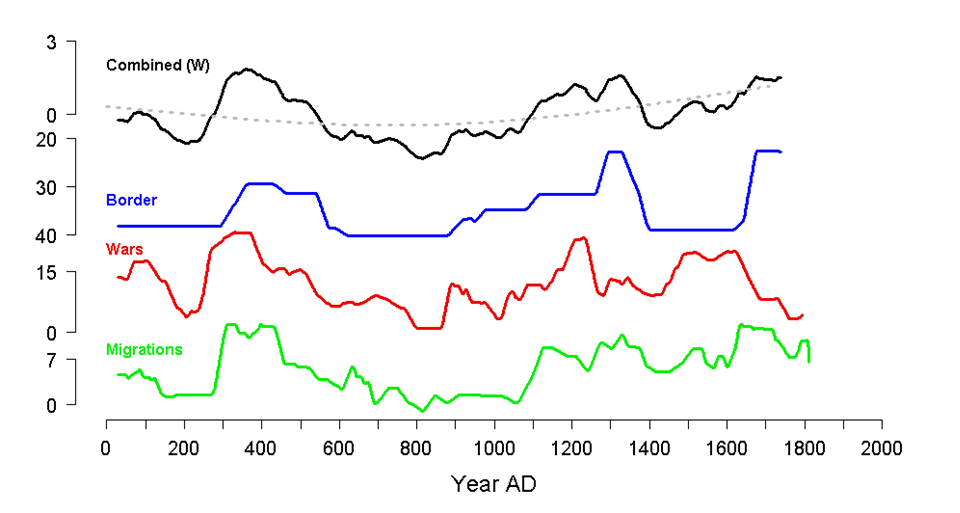


**Fig S10** The War index (W) and its constituent parts; border latitude and the number of years with recorded battles and immigration events per 30 years for nomad pastoralists vs. Han Chinese. The data are from Fang and Liu (Fang and Liu 1992) Fig. 2, linearly extrapolated and smoothed over 30-year moving averages. The combined index W is the sum of the normalized series for war, migration and border distance from of 20ºN. The detrending line used for calculating the cross-correlation function with the climate forcing (Y) on plague (see below) is shown in grey.


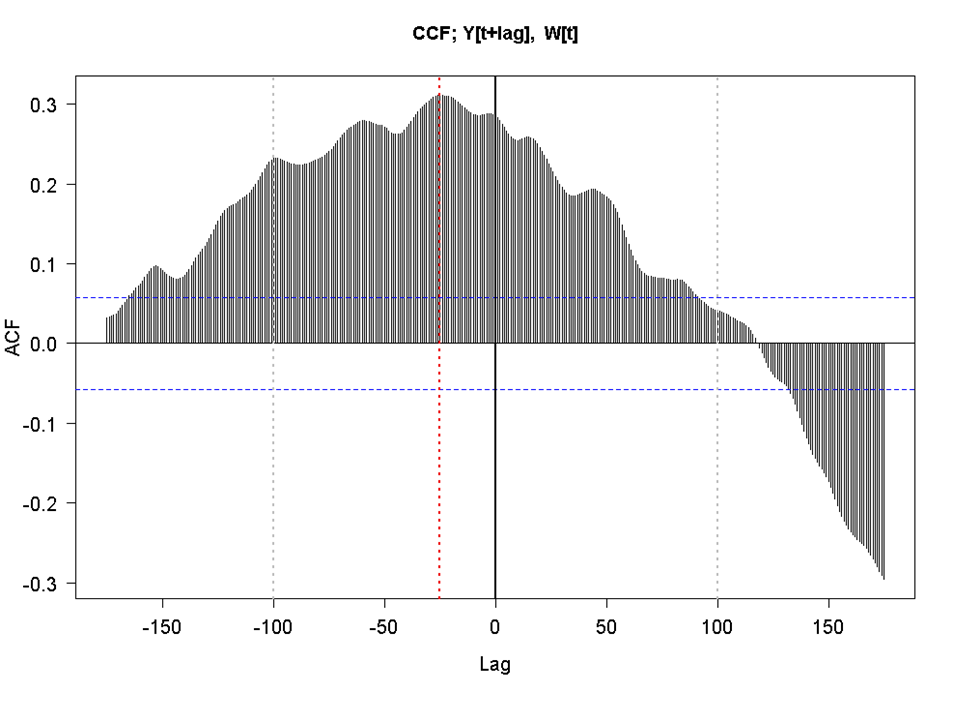


**Fig S11** Cross-correlation function for climate forcing plague estimate (Y) on detrended war frequency (W) for time lags up to 175 years. We see that the correlation is strongest for immediate to centennial scales, with the maximum at a time lag of 25 years, i.e. around one human generation.

***Statistical methods***

***Climate forcing on metapopulation dynamics -effects of scale***

A large-scale climate signal X can have a forcing effect on a set of *i* spatially separated sub-populations linked by local migration. For the sake of these analyses, we consider the case of discrete-time model dynamics on an annual scale. Here, the size of each sub-population, N_1-i_, is influenced by endogenous dynamics (density dependence) and migration as well as local exogenous forcings Z_1-i_ unrelated to the larger-scale signal. The correlation between X and population size will then be scale dependent in time and space. In space, the population size of single sub-populations will tend to be less correlated with the exogenous signal than the mean $\overline{N}$of n sub-populations.

$\overline{N}=\frac{1}{n}\sum_{i=1}^{n} N_{i}$ (S1)

This can be illustrated through the simple case of 200 populations being linked in one dimension through dispersal so that a proportion η migrates annually from each population to its two neighbors. Scaling the external forcing signals so that X~Normal(0,1) and Z_i_~Normal(0,1), and assuming that they are independent between years *t*, we can include both signals as modifiers of death rate ω, birth rate R and carrying capacity K in a population model so that $\omega_{i,t}=\frac{\omega}{1+\exp\left(-X_{t}-Z_{i,t}\right)}, R_{i,t}=\frac{R}{1+\exp\left(-X_{t}-Z_{i,t}\right)}, \text{and} K_{i,t}=\frac{K}{1+\exp\left(-X_{t}-Z_{i,t}\right)}$ (S2-4)

So, assuming a density independent component in survival and a density-dependent population growth rate, we can express the population dynamics of each sub-population as:

$N'_{i,t}=\omega_{i,t}N_{i,t-1}+N_{i,t-1}R_{i,t}\frac{1}{1+\exp\left(k_{0}\left[k_{1}+K_{i,t}-N_{i,t-1}\right]\right)}$ (S5)

Population sizes before dispersal (N') are truncated at zero and then updated by local dispersal expressed as

$N_{i,t}=\frac{1}{2}\eta N'_{i-1,t}+\frac{1}{2}\eta N'_{i+1,t}+(1-\eta)N'_{i,t}$ (S6)

One run of the model with 200 populations is shown in Fig. S12a. Neighbouring sub-populations are tightly linked through migration, but the correlation decays rapidly with distance until it reaches the level where distance does no longer matter but a non-zero correlation is maintained through the shared forcing component (X) (Fig. S12b).

We then see that the correlation between the exogenous signal X and measured population size to some extent depend on the number of populations sampled (Fig. S12c). Sampling only one or a few populations give a lower correlation between $\overline{N}$ and X as the local endogenous dynamics are relatively strong compared to the exogenous forcing. Increasing the number of populations sampled will, up to a saturation point, show a stronger correlation between $\overline{N}$ and X, as the impact of the shared climate signal tend to synchronize populations, but while phase effects are averaged out due to local stochasticity (Fig. S12c). Likewise, the correlation between total average population size and external forcing may depend on the temporal scale at which we measure X. Despite X only having instantaneous effects in the model and being itself independently variable between years, the system integrates over more years, so the best correlation between the forcing signal X and the large-scale population average $\overline{N}$ is found by taking the moving average of X over of *t* years,

$\overline{X}=\frac{1}{t}\sum_{i=1}^{t} X_{i}$ (S7)

It then becomes apparent that depending on population growth rates, the temporal frequency (window length) at which climate forcing is reflected most strongly in population size can be significantly greater than one year, even in this simple system (Fig. S12d), and is dependent on stochastic effects.


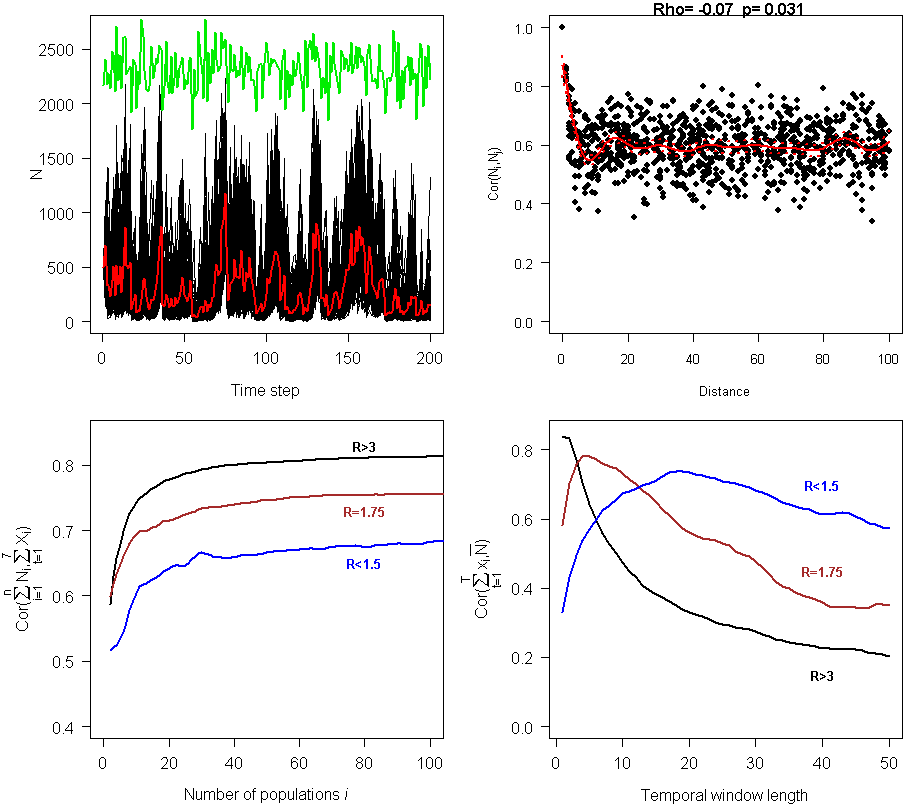


**Fig S12** **a)** A run of the stochastic model in eq. S2-S6. The green line is the climate signal X, while the black lines show all sub-populations, and the red line their average ($\overline{N}$). **b)** Non-parametric correlogram showing the correlation between sub-populations as a function of the distance between them. Even though dispersal only happen between neighboring populations (distance=1) , correlation is stronger than average for greater distances (in this case up to distances of about five populations). **c)** Correlation between a moving average of the climate signal (using optimum window lengths for the value of R) and the average of *i* sub-populations. We see that for all different values of R the climate signal is most visible when at least twenty sub-populations are averaged over. **d)** Correlation between moving averages of different lengths for X and the mean total population size, showing the increase in optimum window length for decreasing R, and that the strongest correlations between climate forcing and population size can be found at lower frequencies.

We therefore conclude that when a number of locally linked populations are affected by the same climate fluctuations, the effect is relatively stronger on larger scales spatially (summing over several sub-populations) as well as temporally (lower frequencies). The temporal scale at which densities are most coherent with external forcing depend on the endogenous dynamical properties of the populations, even when there is no temporal autocorrelation in the climate fluctuations.

***On shared signals and model selection***

Here we are concerned with the densities of plague host and vector populations across the arid areas of Central Asia that experience inter-annual climate variability correlated with that of Eastern Kazakhstan (PreBalkhash). It seems reasonable to suspect that this natural system, where a large number of locally dispersing populations (burrow-dwelling rodents, with their pathogens and parasites) are affected by a large-scale climate influence (annual variation in precipitation and temperature being highly correlated over large areas of Central Asia) corresponds to the principles discussed above. If so, the relative strength of the system's response to external (climate) forcing as opposed to endogenous dynamics (density dependence) is strongest on large spatial scales and on lower than annual frequencies. Thus, as we are concerned with large-scale changes in plague abundance, a climate-driven statistical model may be suitable for reflecting large-scale changes.

We therefore approach the large-scale average plague abundance (product of host density and plague prevalence) as a signal in principle consisting of the sum of the forcing effects captured by the climate data, and effects unrelated to these (i.e. endogenous dynamics and exogenous forcing not captured in the available data). As human plague cases ultimately originate in the host reservoirs, the number of annual human cases should be related to the zoonotic plague abundance. However, few assumptions can be made about the shape and frequency of the effect of climate on plague abundance, and of plague abundance on human cases. Thus, an analytical solution to the joint probabilities of the different relationships is intractable without simplifying assumptions that are likely to be unwarranted. But we can test the validity of our methodological approach through a simplified simulation experiment.

Let us have a climate time series X which reflects processes with a forcing effect on large-scale plague abundance β. Variation in this zoonotic plague abundance causes corresponding variation in the frequency of human plague cases γ. However, for the reasons discussed above, the main effect of X on β may take place on a lower-than-annual frequency Z_β_. In addition, both random events, unknown endogenous dynamics and external forcing not captured by the time series X also affect plague abundance.

Thus, β can be seen as consisting of a moving average of X of length Z_β_ plus a "noise" component ε_β_ that has no regular distribution and contains both white and red noise. Likewise, γ can be seen as being a (possibly non-linear) function of β and another "noise" component ε_γ_ that contains randomness and processes unrelated to β.

According to our methodology, if we use a model selection criterion like AIC or GCV to score statistical models of β as a function of X at different frequencies (Z_β_ being "unknown"), we should find that the models with the lowest scores perform best at predicting the values of β for parts of the time series that has not been used to train the model (Akaike et al. 1976). If the impact of the climate signal is very low, it of course has no predictive power regarding either β nor γ. However, if there is a real and consistent relationship between X and β, and between β and γ, we should expect the models that best predict β to also predict γ for the withheld parts of the time series. The degree to which good models of β also are good predictors of γ obviously will depend on the strength of the association between β and γ in the first place.

Let us consider a case where $X\sim Normal(0,1)$, with no temporal autocorrelation, as this seems a reasonable approximation to a measured climate component such as annual temperature. But so as to make no unwarranted assumptions about the structure of the "unobserved" contributions to the processes, we assign them a mix of Normal and Poisson distributions with variable frequencies, so that

$\varepsilon_{\beta,t}=\sum_{i=t}^{t-z_{\varepsilon\beta}} Poisson(\lambda_{\beta})_{i}+Normal(0,1)$ (S7)

and

$\varepsilon_{\gamma,t}=\sum_{i=t}^{t-z_{\varepsilon\gamma}} Normal(0,1)_{i}+Poisson(\lambda_{\gamma})$. (S8)

Choosing a frequency component Z_β_ we get a signal β representing the plague abundance, where the values of the constants λ and Z govern the signal-to-noise ratios and frequencies:

$\beta_{t}=\sum_{i=t}^{t-z_{\beta}} X_{i}+\varepsilon_{\beta,t}$ (S9)

Then, we let the anomalies in the frequency of human cases γ be a non-linear function of β:

$\gamma_{t}=\frac{g_{0}}{1+\exp(\beta_{t}-g_{1})}+\varepsilon_{\gamma,t}$ (S10)

We then simulate the situation of there being an unknown statistical relationship between X and β by fitting a set of generalized additive models (GAMs), for different values of z to test the effect of X on different frequencies:

$\beta_{t}=f(M_{mean}(X_{t},z))+\varepsilon_{t}$ (S11)

The AIC is recorded for each model fit (AIC being used here instead of the GCV due to the Gaussian errors in S11).

We then run the simulation 10^2^ times to test for sensitivity to different noise frequency parameter values (Z_εβ_ and Z_εγ_ in eqs. S7 and S8) and random permutations of the stochastic variables (X and the random components of eqs. S7 and S8). We use a time series length of 100 years, fitting the statistical models (eq. S11) on 50 years and predicting the 50 witheld years, analogous to our main analysis. The results suggests that the AIC consistently selects models that picks up the true value of Z=Z_β_ and good estimation of non-linearities. Moreover, as AIC decreases, the correlation between the predicted β-values and their corresponding γ-values are consistently increasing, on average asymptotically approaching the strength of the true association between β and γ (Fig S13).


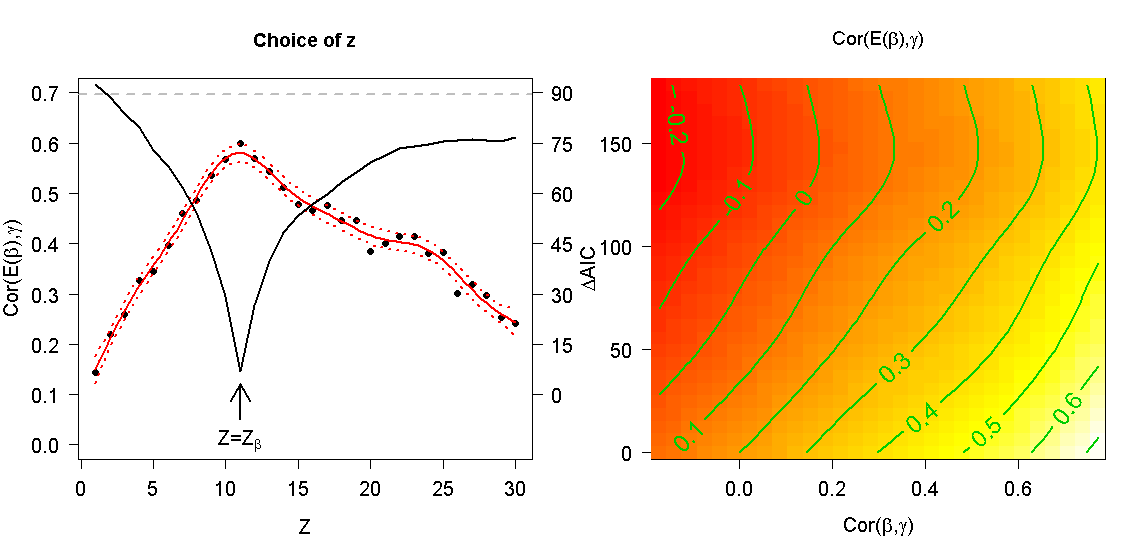


**Fig S13 a**) The black line shows ΔAIC for models (eq. S11) using different values of z, showing a minimum for the true value Z=Z_β_. The red smoothing line shows the correlation between the predicted values of β for the withheld parts of the time series and the values of γ for the same period. We see that this correlation also peaks for the optimum model, approaching the true correlation of (in this case) r=0.7. b) Results for 10^2^ simulatin runs, showing the expected relationship between the true correlation between β and γ, ΔAIC and the correlation between predicted β and γ for the withheld part of the time series. As expected, we see that when β and γ are really correlated, the models selected by AIC for β also predict γ.

In conclusion, we find that the principle behind our modeling approach seems supported. Hidden dependencies in the data and model structure may of course make it statistically invalid in our more complex case, but the results are at the very least consistent with the hypothesis that climate fluctuations have played a role in the epidemiological history of plague.

## References (Supporting Information)

Akaike H, Raman KM, Dimitri GL (1976) Canonical Correlation Analysis of Time Series and the Use of an Information Criterion. In: Mathematics in Science and Engineering, vol Volume 126. Elsevier, pp 27-96

Fang J-Q, Liu G (1992) Relationship between climatic change and the nomadic southward migrations in eastern Asia during historical times. Climatic Change 22:151-168
